# Melatonin, a natural antioxidant therapy in spinal cord injury

**DOI:** 10.3389/fcell.2023.1218553

**Published:** 2023-08-25

**Authors:** Lei Xie, Hang Wu, Xiaohong Huang, Tengbo Yu

**Affiliations:** ^1^ Institute of Sports Medicine and Health, Qingdao University, Qingdao, China; ^2^ Department of Orthopedic Surgery, Qingdao Hospital, University of Health and Rehabilitation Sciences (Qingdao Municipal Hospital), Qingdao, China; ^3^ Department of Orthopedic Surgery, The Affiliated Hospital of Qingdao University, Qingdao University, Qingdao, China; ^4^ Shandong Institute of Traumatic Orthopedics, Medical Research Center, The Affiliated Hospital of Qingdao University, Qingdao, China

**Keywords:** melatonin, spinal cord injury, oxidative stress, melatonin receptor, secondary injury inflammatory response, cell death

## Abstract

Spinal cord injury (SCI) is a sudden onset of disruption to the spinal neural tissue, leading to loss of motor control and sensory function of the body. Oxidative stress is considered a hallmark in SCI followed by a series of events, including inflammation and cellular apoptosis. Melatonin was originally discovered as a hormone produced by the pineal gland. The subcellular localization of melatonin has been identified in mitochondria, exhibiting specific onsite protection to excess mitochondrial reactive oxygen species and working as an antioxidant in diseases. The recent discovery regarding the molecular basis of ligand selectivity for melatonin receptors and the constant efforts on finding synthetic melatonin alternatives have drawn researchers’ attention back to melatonin. This review outlines the application of melatonin in SCI, including 1) the relationship between the melatonin rhythm and SCI in clinic; 2) the neuroprotective role of melatonin in experimental traumatic and ischemia/reperfusion SCI, i.e., exhibiting anti-oxidative, anti-inflammatory, and anti-apoptosis effects, facilitating the integrity of the blood–spinal cord barrier, ameliorating edema, preventing neural death, reducing scar formation, and promoting axon regeneration and neuroplasticity; 3) protecting gut microbiota and peripheral organs; 4) synergizing with drugs, rehabilitation training, stem cell therapy, and biomedical material engineering; and 5) the potential side effects. This comprehensive review provides new insights on melatonin as a natural antioxidant therapy in facilitating rehabilitation in SCI.

## 1 Spinal cord injury

### 1.1 Oxidative stress following spinal cord injury

Spinal cord injury (SCI) is a sudden onset of disruption to the spinal neural tissue, e.g., spinal cord compression caused by accidents or tumors (Baptiste and Fehlings, 2006), leading to paraplegia, quadriplegia, pain hypersensitivity, and abnormal temperature sensation (Khorasanizadeh et al., 2019; Anjum et al., 2020; Eli et al., 2021). Moreover, a series of complications caused by SCI, such as neurogenic bladder, gastrointestinal dysfunction, and sexual dysfunction, decrease the quality of life of SCI patients, which brings a huge burden to society (Popa et al., 2010; Tollefsen and Fondenes, 2012; Sezer et al., 2015; Tate et al., 2020). Though the initial trauma in SCI is irretrievable, preventing adverse events brought by the secondary injury cascade over the subsequent weeks is achievable.

Oxidative stress is a hallmark of secondary injury. It exists from the acute to chronic phases after traumatic and ischemia/reperfusion SCI (Erten et al., 2003; Song et al., 2013). Briefly, the balance between pro-oxidants and antioxidants is disrupted, excessive reactive oxygen species (ROS) are generated, and lipid peroxidation happens, which results in oxidative stress (McDonald and Sadowsky, 2002). The neurons and glial cells in the spinal cord cannot resist the oxidative stress, and cell death consequently happens followed by large amounts of debris (Bains and Hall, 2012). The deteriorated microenvironment initiates secondary injuries, e.g., neuroinflammation and cellular apoptosis (Wang H. et al., 2022).

### 1.2 Melatonin rhythm is affected in spinal cord injury

Primary sleep disorders, like circadian rhythm sleep–wake disorders and insomnia, are commonly encountered in SCI patients, especially in patients with quadriplegia (Zeitzer et al., 2000). Attributing to the role of melatonin (N-acetyl-5-methoxy tryptamine) in regulating the circadian rhythm sleep–wake cycle (Vasey et al., 2021), Zeitzer et al. (2000) first reported that plasma melatonin concentration failed to increase at night in three lower cervical SCI patients in the year 2000 (Zeitzer et al., 2000). Afterward, the temporal dynamics of melatonin has been measured in SCI patients with various injury degrees at different segments (Table 1). Notably, melatonin secretion decreases and rhythm loss occurs in the tetraplegic patients with complete cervical SCI, while they are not affected in complete thoracic SCI (Zeitzer et al., 2000; Scheer et al., 2006; Verheggen et al., 2012; Thøfner Hultén et al., 2018). A melatonin replacement therapy was proposed by Scheer et al. (2006) to improve sleep quality in tetraplegic patients (Scheer et al., 2006). Paralleled with the clinical reports, a similar pattern of changes in the melatonin rhythm has been revealed in the rat SCI model (Table 1) (Gezici et al., 2010). It has been reported that the rhythmic secretion of melatonin from the pineal gland is regulated by the suprachiasmatic nucleus (SCN) in the anterior hypothalamus (Cardinali and Pévet, 1998). The efferent nerve fibers from the SCN to the pineal gland cross the upper part of the cervical medulla and are connected to the preganglionic cells of the cervical sympathetic ganglion (Møller and Baeres, 2002), which hints the causality of patients with SCI at or above C8 with inadequate melatonin secretion, disrupted melatonin rhythm, and poor sleep quality (Verheggen et al., 2012). However, patients with injuries below the T4 spinal cord maintain their melatonin rhythm (Whelan et al., 2020).

**TABLE 1:** Relationship between the temporal dynamics of melatonin and spinal cord injury in patients and animal models.

No.	Subject	Segment	Melatonin			Ref.
			Sampling	Concentration	Rhythm	
1	Patient	3C	Plasma	Decreases at day and night compared to healthy controls	Absent	Zeitzer et al. (2000)
		2T		Similar to healthy controls	Present	
2	Patient	3C	Plasma	Decreases at day and night compared to healthy controls	Absent	Scheer et al. (2006)
		2T		Similar to healthy controls	Present	
3	Patient	6C	Saliva	Fails to increase at night compared to healthy controls	Absent	Verheggen et al. (2012)
		9T		Similar to healthy controls	Present	
4	Patient	13C	Saliva	Decreases at night compared to healthy controls	Absent	Thøfner Hultén et al. (2018)
		6T		Similar to healthy controls	Present	
5	Rat	10C	Serum	Decreases at night compared to that of the sham rats	Absent	Gezici et al. (2010)
		10T		Similar to sham rats	Present	

C, cervical; T, thoracic.

## 2 Melatonin and melatonin receptors

### 2.1 Melatonin

Melatonin is an amphiphilic molecule first isolated by Lerner et al. (1958). Melatonin is synthesized in the pineal gland in the central nervous system (CNS) and peripheral organs, including the retina, intestine, bone marrow, pancreas, and kidney (Reiter, 1991; Ren et al., 2017; Zhang Y. et al., 2018). The subcellular localization of melatonin has been identified in mitochondria due to that the melatonin synthetic enzyme serotonin N-acetyltransferase (SNAT) was found in the matrix and intermembrane space of mitochondria (Reiter and Tan, 2019). Mitochondria are the major source of ROS, the birth place and battle ground of melatonin, and also the site of melatonin metabolism (Reiter and Tan, 2019). Melatonin exhibits specific on-site protection against mitochondrial ROS, which is underlying melatonin’s anti-oxidative mechanism. Reiter and Tan (2019) proposed that melatonin exhibits its major protective effects on neurons at the level of mitochondria (Reiter and Tan, 2019). Melatonin receptor (MT1) is located on the mitochondrial membrane, and it regulates mitochondrial physiology through the MT_1_-mediated sirtuin 1 (SIRT1)– peroxisome proliferator-activated receptor-gamma coactivator-1 alpha (PGC-1α)–nuclear factor erythroid 2–related factor 2 (NRF2)– peroxisome proliferator-activated receptor-gamma (PPAR-γ) signaling transduction through the MT_1_-mediated sirtuin 1 (SIRT1)– peroxisome proliferator-activated receptor-gamma coactivator-1 alpha (PGC-1α)–nuclear factor erythroid 2–related factor 2 (NRF2)– peroxisome proliferator-activated receptor-gamma (PPAR-γ) signaling transduction (Guo et al., 2014). It has been reported that melatonin maintains mitochondrial homeostasis by regulating mitochondrial fission, mitophagy, and mitochondrial permeability transition pore (Zheng et al., 2021). Specifically, mitochondrial ROS in cancer amplifies the tumorigenic phenotype, accelerates the accumulation of additional mutations, and leads to metastatic behaviors (Sabharwal and Schumacker, 2014); the cytotoxicity of melatonin to Caco-2 cells (an immortalized cell line of human colorectal adenocarcinoma cells) and Jurkat cells (an immortalized cell line of human T lymphocyte cells), in turn, evidences the effectiveness of melatonin in regulating the mitochondrial metabolism (Wölfler et al., 2001).

Plasma melatonin concentration peaks at night in mammals (Zhang et al., 2022). It has been suggested by experts that the exposure to Sun during the day and avoidance of light at night benefit human wellbeing by improving melatonin production (Tan et al., 2023). In clinic, melatonin has been used to treat primary insomnia (Rondanelli et al., 2011). Moreover, the effectiveness of melatonin in combating oxidative stress, inflammation, and cellular apoptosis has been studied for decades in mood disorders and cognitive dysfunctions (Agahi et al., 2018), stroke and other neurodegenerative diseases (Hosseinzadeh et al., 2023), tumors, and cancers (Rohilla et al., 2023) Recently, the cryogenic electron microscopy (cryo-EM) structures of the active melatonin receptors (MTs) have been revealed (Wang Q. et al., 2022), which is going to take melatonin research forward.

### 2.2 Melatonin receptors and melatonergic ligands

Melatonin regulates sleep and the circadian rhythm by activating two high-affinity G protein–coupled receptors, i.e., MT_1_ and MT_2_ (Cecon et al., 2018). In the brain, MT_1_ is expressed in the locus coeruleus (LC) and perifornical lateral hypothalamus (PFH), regulating the rapid eye movement (REM), while MT_2_ is found in the thalamic reticular nucleus (TRN) in charge of the non-REM (NREM) activity (Lacoste et al., 2015; Waly and Hallworth, 2015). Initially, melatonin is extracted from the pineal gland and urine; currently, all melatonin used in clinic is obtained from chemical methods, such as the prolonged melatonin preparation (Circadin^®^) and the synthesized ligand agonist to both MT_1_ and MT_2_ (agomelatine, ramelteon, and tasimelteon) (Wang Q. et al., 2022). The technological breakthrough, obtaining biological melatonin from microbes, algae, and plants, has been achieved a few years ago (Arnao et al., 2023). Notably, the microorganisms, including the genetically modified *Saccharomyces* and *Escherichia coli* and microbial fermentation *Lactobacillus* sp., *Bifidobacterium* sp., *Enterococcus* sp., and *Streptococcus thermophilus*, haven been employed to produce melatonin industrially in bioreactors (Arnao et al., 2023). Despite the development of the chemical and biological melatonergic ligands, the *in vivo* stability and subtype selectivity, especially the MT_1_ selective drug, are yet to be solved (Boutin et al., 2020). From the pharmacological standpoint, a full characterization of the melatonin receptor subtypes is advocated. Wang H. et al. (2022) revealed that the cryo-EM structures of active MTs with heterotrimeric G_i_ protein engaged in the receptor core (Wang Q. et al., 2022). Though MT_1_ and MT_2_ possess a high similarity in the orthosteric ligand-binding pockets, they display distinctive features in the cryo-EM, i.e., the distinctive structure of the N4.60-Y5.38-H5.46 motif and the “longitudinal channel” in MT_1_ and the larger sub-pocket in MT_2_ (Wang Q. et al., 2022).

Attributing to the relationship between the melatonin rhythm and SCI in clinic (Table 1), efforts have been paid to bond the causality between rehabilitation and melatonin in SCI. To provide a thorough summary of melatonin and its effect associated with SCI, literature searches were conducted on the databases of PubMed, Web of Science, and Google Scholar from inception to March 2023 with the keywords combining “spinal cord injury” or “SCI” with “melatonin” or “N-acetyl-5-methoxy tryptamine.” The eligibility criteria of the selected studies are 1) relevant studies with full text published in English and assessable online; 2) outcomes obtained from animal models (mouse, rat, and rabbit); 3) reporting information including model type, treatment, dosage/duration of melatonin, and outcome assessment; and 4) modeling with contusion, compression, radiation, photo thrombosis, and spinal cord ischemia–reperfusion injury (SCIR).

## 3 Melatonin confers neuroprotection in spinal cord injury

The neuroprotective effects and underlying mechanisms of melatonin were explored in experimental SCI (Table 2).

**TABLE 2:** Non-comprehensive review of the neuroprotective effects of melatonin in experimental spinal cord injury.

No.	Animal (gender/weight)	Model	Control	Treatment	Melatonin (dosage/duration)	Assessment	Major findings	Ref.
1	C57BL/6 mouse (M, 22–30 g)	T9 weight-drop	Sham SCI	SCI + melatonin (50 mg/kg, i.p.)	2 hpi and qd for 7 dpi	BMS (1, 3, 7, 14, 21, and 28 dpi)	Melatonin promotes neurons survival and accelerates the recovery of nerve function	Guo et al. (2023)
							Melatonin promotes M2 polarization of microglia and decreases pro-inflammatory-related mRNA levels	
							Melatonin attenuates ROS activity and recovers mitochondrial metabolism	
2	C57BL/6 mouse (M and F, 20–25 g)	T9–10 weight-drop	Sham SCI + V	SCI + melatonin (30 mg/kg, i.p.) SCI + melatonin (30 mg/kg, i.p.) +ML385	0, 12, and 24 hpi	BMS (1, 3, 5, 7, 10, 14, 21, 28 dpi)	Melatonin prevents motor neurons apoptosis and improves the recovery of nerve function	Wang et al. (2022a)
							Melatonin reduces ROS and mitochondrial damage	
							Melatonin positively regulates NRF2, which activates the ARE signaling transduction and suppresses the NLRP3 inflammasome	
3	SD rat (ND, ∼250 g)	T9 weight-drop	Control SCI + luzindole	SCI + melatonin (15 mg/kg, i.p.)	ND	BBB, tail tenderness (1, 3, 7, and 14 dpi)	Melatonin accelerates rehabilitation	Bi et al. (2021)
							Melatonin reduces neuron apoptosis and promotes neuronal repair	
							Melatonin promotes synaptic remodeling via activating the PI_3_K–AKT1 pathway	
4	Wistar rat (M, 230–270 g)	T6–7 clamp compression	Sham SCI + V	SCI + melatonin (0.4 umol, intrathecal)	30 mpi	Von frey test, acetone drop test, inclined plane test, BBB, weight change, and auricle temperature (7, 14, 21, and 28 dpi)	Melatonin ameliorates motor dysfunction, mechanical and cold allodynia, auricle temperature, and weight loss	Fakhri et al. (2021)
							Melatonin preserves the white matter-myelinated areas and maintains the number of sensory neurons	
							Melatonin exhibits anti-neuroinflammatory effects via suppressing the activity of MMP9 while increasing that of MMP2 post SCI	
5	Wistar rat (M, 250–300 g)	T6 weight-drop	Control sham SCI	SCI + melatonin (10 mg/kg, s.c.)	0, 12, 24, 36, 48, 60, and 72 hpi	BBB (1, 2, and 3 dpi)	Melatonin decreases axonal degeneration and demyelination	Majidpoor et al. (2020a)
							Melatonin reduces the formation of NLRP1, NLRP3, and AIM2 inflammasome complexes	
							Melatonin suppresses the activity of the TLR4-mediated NF-κB and NOX2/TXNIP signaling pathways	
6	Wistar rat (M, 250–300 g)	T1, T6, and T12 weight-drop	Control sham SCI	SCI + melatonin (10 mg/kg, s.c.)	0, 12, 24, 36, 48, 60, and 72 hpi	BBB (3 dpi)	Melatonin promotes the recovery of spinal cord function	Majidpoor et al. (2020b)
							NLRP3 inflammasome is increased regardless of the segment of SCI	
							Melatonin decreases NLRP3 inflammasome and axonal demyelination	
7	SD rat (M, 260–300 g)	T9–10 weight-drop	Sham SCI + V	SCI + melatonin (10 mg/kg, i.p.) SCI + melatonin (10 mg/kg, i.p.) + EX527 (SIRT1 inhibitor)	0, 12, and 24 hpi	BBB (1, 3, 7, 14, 21, 28, 35, and 42 dpi)	Melatonin promotes the recovery of motor function in the hind limbs 2 weeks after SCI. Melatonin inhibits apoptosis and active autophagy in the nerve cells through the AMPK-mediated signaling pathway via activating SIRT1	Gao et al. (2020a)
8	C57BL/6 mouse (M, 22–25 g)	T11 clamp compression	Sham SCI + V	SCI + melatonin (10 mg/kg, i.p.)	1 hpi and qd for 7 dpi	BMS (Qd for 7 dpi)	Melatonin facilitates functional recovery	Yang et al. (2020)
							Melatonin suppresses the accumulation and proliferation of microglia and astrocytes	
							Melatonin reduces the release of pro-inflammatory cytokines	
							Melatonin attenuates oxidative stress	
9	SD rat (M, 240–260 g)	T10 weight-drop	Sham SCI + V	SCI + melatonin (12.5 mg/kg, i.p) SCI + melatonin (12.5 mg/kg, i.p) +3-MA	Qd for 7 dpi	BBB (1, 3, 7, 14, and 28 dpi)	Melatonin improves the recovery of locomotor function	Li et al. (2019)
							Melatonin increases the number of survival neurons indicated by Nissl staining	
							Melatonin enhances autophagy and reduces apoptosis by activating the PI_3_K/AKT signaling pathway	
10	Wistar rat (F, 220–250 g)	T10 weight-drop	Sham SCI + V	SCI + melatonin (50 mg/kg, i.p.)	0 hpi and qd for 7 dpi	BBB (Qd for 1 dpi)	Melatonin promotes functional recovery in SCI rats	Zhang et al. (2019)
							Melatonin promotes M2 polarization of microglial/macrophages during the early stage of SCI	
							Melatonin reduces neuron apoptosis and inhibits pro-inflammatory responses	
11	SD rat (ND, 180–220 g)	T9–10 weight-drop	Sham + V SCI + V	SCI + melatonin (12.5 mg/kg, i.p.)	Qd for 10 dpi	BBB (1, 3, 7, 14, 21, and 28 dpi)	Melatonin accelerates rehabilitation via mitigating cellular apoptosis	Xu et al. (2018)
							Melatonin inhibits NLRP3 expression, suppressing the activation of inflammasomes	
12	SD rat (F, 180–220 g)	T9–10 weight-drop	Sham + V SCI + V	SCI + melatonin (12.5 mg/kg, i.p.)	1, 2, 3 dpi	BBB (1, 3, 7, 14, 21, and 28 dpi)	Melatonin improves functional recovery after SCI	Shen et al. (2017)
							Melatonin reduces neuronal apoptosis indicated by the reduced TUNEL-positive cells, but increases the expression of BCL2	
							Melatonin attenuates SCI by activating the wingless–int/β-catenin signaling	
13	WT C57BL/6 mouse (ND, 20–22 g)	T6–7 clamp compression	Sham + V sham + melatonin SCI	SCI + melatonin (30 mg/kg, i.p.)	1, 6, and 12 hpi	BMS (Qd for 10 dpi)	Melatonin facilitates rehabilitation in SCI. Melatonin reduces inflammation and tissue injury, neutrophil infiltration, pro-inflammatory cytokine expression, NF-κB activation, and iNOS expression. Melatonin mediates anti-inflammatory activity by activating PPAR-α	Paterniti et al. (2017)
	PPAR-α KO C57BL/6 mouse (ND, 20–22 g)		Sham + V sham + melatonin SCI	SCI + melatonin (30 mg/kg, i.p.)				
14	SD rat (F, 180–220 g)	T10 weight-drop	Sham SCI + V	SCI + melatonin (10 mg/kg, i.p.)	30 mpi and bid for 7 dpi (07:00, 19:00)	ND	Melatonin restrains blood vessel loss and ameliorates BSCB permeability	Jing et al. (2017)
							Melatonin increases neuron number and Nissl bodies within neurons at the injury epicenter	
							Melatonin facilitates the synaptic remodeling in the spinal cord and hippocampus	
15	Mice (F, ND)	T12 clamp compression	Sham SCI	SCI + melatonin (10 mg/kg, i.p.)	Single dose: 10 mpi; 14 daily doses: 10 mpi; and qd for 14 dpi	BMS (1, 3, 5, 7, 10, and 14 dpi)	Melatonin increases the expression of axon/neuronmarkers	Krityakiarana et al. (2016)
							Melatonin treatment for 14 days reduces inflammatory response and scar formation	
16	SD rat (F, 180–220 g)	T10 weight-drop	Sham SCI + V	SCI + melatonin (50 mg/kg, i.p.)	30 mpi and bid for 7 dpi (07:00, 19:00)	ND	Melatonin ameliorates spinal cord perfusion in regions rostral and caudal to the injury site, rather than the epicenter site	Jing et al. (2016)
							Melatonin upregulates spinal cord blood flow and oxygen saturation in regions of rostral and caudal cords	
							Melatonin reduces the amount of Evan’s blue dye and water content in regions of rostral and caudal cords	
17	SD rat (M, 205–225 g)	T12 clamp compression	Sham SCI + saline	SCI + melatonin (100 mg/kg, i.p.)	0 hpi and qd for 2 dpi	ND	Melatonin eliminates astrocytic swell and reduces spinal cord edema via downregulating the protein expression of AQP4	Liu et al. (2015)
18	C57BL/6 mouse (M, 20–25 g)	T10 weight-drop	Sham + V SCI + V	SCI + melatonin (5, 10, 25, 50, and 100 mg/kg, i.p.)	0 hpi	ND	Melatonin maintains the microcirculation and attenuates edema, protecting the tight junction proteins, endothelial cells, and pericytes	Wu et al. (2014)
							Melatonin decreases cellular apoptosis	
							Melatonin reduces the expression of MMP3, AQP4, HIF1α, VEGF, and VEGFR2	
19	SD rat (F, 228–272 g)	T12 clamp compression	Sham SCI + saline	SCI + melatonin (100 mg/kg, i.p.)	0 hpi and qd for 2 dpi	ND	Melatonin alleviates spinal cord edema	Li et al. (2014)
							Melatonin decreases AQP4 and GFAP	
20	C57BL/6 mouse (M, 18–22 g)	T10 weight-drop	Sham SCI + V	SCI + melatonin (10 mg/kg, i.p.)	Bid for 14 dpi (07:00, 19:00)	BMS and subscore (2, 7, 10, and 14 dpi)	Melatonin rescues the motor neurons and promotes the recovery of locomotor function	Wu et al. (2014)
							Melatonin increases the pericyte coverage, ameliorates blood vessel loss, and alleviates BSCB disruption	
							Melatonin upregulates ANGPT1, inhibiting inflammation and apoptosis in pericytes	
21	Wistar rat (M, 250–300 g)	T9 compression 35% (0.78-mm thick spacer) and 50% (1.11-mm thick spacer)	Sham + V sham + melatonin 50% SCI + V 35% SCI + V	35% SCI + melatonin (2.5 mg/kg, i.p.) 50% SCI + melatonin (2.5 mg/kg, i.p.)	5 min pre and 1, 2, 3, and 4 hpi	Open-field test, BBB, inclined plane test (1, 7, 14, 21, 28, and 35 dpi)	Melatonin treatment facilitates functional recovery	Schiaveto-de-Souza et al. (2013)
							Melatonin facilitates the integrity of the spinal cord thoracic segment and alleviates the severity of the lesion after spinal cord narrowing	
22	Mice (ND)	T6–7 clamp compression	Sham + saline sham + melatonin	SCI + melatonin (50 mg/kg, i.p.)	0, 1, 6, and 12 hpi	BBB (Qd for 20 dpi)	Melatonin improves motor recovery	Esposito et al. (2009)
							Melatonin administration leads to a reduction in the MAPK signaling pathway, indicated by the decreased expression of MAPKs p38, JNK, ERK1/2, and TNF-α	
							Melatonin decreases the expression of HMGB1	
23	Wistar rat (M, 200–250 g)	T4–5 clamp compression	Control SCI + saline SCI + octreotide	SCI + melatonin (7.5 mg/kg, i.p.)	0, (a) 24 hpi or (b) three equal doses over 10 days to achieve 7.5 mg/kg/day	ND	Melatonin decreases the ROS level and reduces necrosis and degeneration in both the initial and late stages of SCI.	Erol et al. (2008)
							Melatonin is superior to octreotide with respect to the prevention of congestion, edema, axonal degeneration, and necrosis	
24	SD rat (M, ND)	T10 weight-drop	Sham sham + melatonin SCI + V	SCI + melatonin (45 mg/kg, i.p.)	15 mpi	ND	Melatonin reduces inflammation, axonal damage, and neuronal death	Samantaray et al. (2008)
							Melatonin attenuates the SCI-induced upregulated activity in CAPN1 and CASP3	
25	SD rat (M, 225–250 g)	T10 weight-drop	Sham SCI + V	SCI + melatonin (10 mg/kg, i.p.)	Qd for 14 dpi or 35 dpi	BBB (1–14, 21, 28, and 35 dpi)	AQP1 expression is increased in survival neurons and reactive astrocytes, which results in neuronal and astrocytic swelling	Nesic et al. (2008)
							Melatonin decreases the expression of AQP1 in the dorsal horn sensory afferents, significantly decreasing mechanical allodynia	
26	Mice (ND)	T6–7 clamp compression	Sham + saline sham + melatonin SCI + saline	SCI + melatonin (50 mg/kg, i.p)	30 min pre and 1, 6, and 12 hpi	ND	Melatonin decreases spinal cord edema and prevents white matter lesion	Esposito et al. (2008)
							Melatonin reduces the activity and expression of TNF-α, MMP2, and MMP9	
							Melatonin decreases the levels of MPO and TBARS	
27	Wistar rat (M, 200–250 g)	T7–10 weight-drop	Sham sham + pinealectomy SCI + pinealectomy	SCI + melatonin (100 mg/kg, i.p.) SCI + pinealectomy + melatonin (100 mg/kg, i.p.)	0 hpi	Motor function scale and inclined plane test (7, 14, 21, and 42 dpi)	Reduction in endogenous melatonin has been detected after pinealectomy, which makes rats more vulnerable to trauma	Ates et al. (2007)
							Exogenous melatonin accelerates motor recovery and attenuates tissue lesion	
							Melatonin decreases oxidative stress indicated by the reduced MDA, XO, and NO levels and increased GSH content compared to SCI	
28	Wistar rat (ND, 230–300 g)	T10 clamp compression	Sham SCI + V SCI + MP	SCI + melatonin (50, 100 mg/kg, i.p.)	0 hpi	Painful stimulus, inclined plane test (12 and 24 hpi)	Melatonin shows no dose-dependent effects on lipid peroxidation	Gül et al. (2005)
							Melatonin exhibits neuroprotective effects on axons and myelin sheaths of white matter compared with MP	
							Neuroprotective effects are augmented as the melatonin dose increases	
29	SD rat (M, 300–350 g)	T5–T8 clamp compression	Sham + saline sham + melatonin SCI + saline	SCI + melatonin (50 mg/kg, i.p.)	30 min pre, 0.5, 1, 6, and 12 hpi	Tarlov’s (1–10 dpi)	Melatonin ameliorates limb function recovery, indicated by the evaluated motor recovery score	Genovese et al. (2005)
							Melatonin reduces inflammation, neutrophils infiltration, and apoptosis	
							Melatonin decreases the formation of nitrotyrosine and poly (ADP-ribose)	
30	SD rat (M, 280–300 g)	T12 weight-drop	Sham SCI + saline	SCI + melatonin (100 mg/kg, i.p.)	4 hpi	Tarlov’s, inclined plane test (1, 3, 7, 14, and 21 dpi)	Melatonin improves the Tarlov’s score and inclined plane angle at 7, 14, and 21 days after SCI	Liu et al. (2004)
							Melatonin decreases edema and neutrophil infiltration and prevents neurons death and axons swollen	
							Melatonin reduces the level of MDA	
31	Wistar rat (M, 250–430 g)	T4-5 clamp compression	Sham SCI	SCI + melatonin (10 mg/kg, i.p.) SCI + oxytetracycline SCI + prostaglandin E1	0 hpi	ND	Melatonin reduces oxidative stress in acute spinal cord injury indicated by the suppressed levels of MDA and PON1 and the increased SOD, GSH-Px, and Hcy	Topsakal et al. (2003)
							Melatonin is more powerful than prostaglandin E1 and oxytetracycline in reducing ROS, especially at the fourth hour post injury	
32	Wistar rat (M, 225–280 g)	T12 weight-drop	Sham SCI + saline	SCI + melatonin (2.5 mg/kg, i.p.)	5 mpi and 1, 2, 3, and 4 hpi	Inclined plane test, Tarlov’s (1, 2, 4, 7, 10, 12, 14, 16, 18, and 21 dpi)	Melatonin facilitates the recovery of the damaged spinal cord and motor function	Fujimoto et al. (2000)
							Melatonin reduces the TBARS and MPO activities	
33	SD rat (M, 250–300 g)	T7–8 clamp compression	Control control + melatonin Control + V Sham SCI + V	SCI + melatonin (40 mg/kg, i.p.)	20 min pre- and 1, 2 hpi	ND	Melatonin decreases GSSG content and increases CAT activity in the control group	Taskiran et al. (2000)
							Melatonin supports the antioxidant defense system indicated by the decreased GSSG activity, elevated GSH/GSSG ratio, but increased SOD and CAT activity without reaching statistical significance in the SCI rats	
34	Wistar rat (M, 200–240 g)	T7–10 weight-drop	Sham SCI + V SCI + MP	SCI + melatonin (100 mg/kg, i.p.)	0 hpi	ND	Melatonin treatment decreases MDA and protects neurons, axons, myelin, and intracellular organelles (mitochondrion and nucleus)	Kaptanoglu et al. (2000)
							Melatonin exhibits better neuroprotective effect than MP on neurons and subcellular organelles after secondary injury	
35	SD rat (M, 250–280 g)	T10 weight-drop	SCI + DD SCI + LL	SCI + L/D	No exogenous melatonin	BBB (3, 7, 14, and 28 dpi)	L/D cycle facilitates neuronal differentiation and inhibits primary neuronal death	Hong et al. (2019)
							L/D cycle increases the endogenous melatonin content in the rostrocaudal region, promoting oligodendrogenesis, excitatory synaptic formation, and axonal outgrowth	
							Melatonin precursor NAS activates the TRKB-induced AKT/ERK signaling	
36	SD rat (M, 250–260 g)	T9–10 weight-drop	Control (L/D) control (L/L) SCI (L/D) SCI (L/L)	SCI + melatonin (L/D, 10 mg/kg, s.c.) SCI + melatonin (L/L, 10 mg/kg, s.c.)	Bid for 28 dpi (07:00, 19:00)	BBB (1, 3, 7, 14, 21, and 28 dpi)	Endogenous and exogenous melatonin increase the hind limb motor function, indicated by the elevated BBB score in the melatonin-administrated group in the L/D condition compared with the LL condition	Park et al. (2012)
							Melatonin attenuates the autophagic signaling, indicated by the decreased expression of MAFbx, MuRF1, iNOS, BECN1, and converted LC3 II protein	
37	Rat (ND)	Infrarenal aorta temporarily occluded	Control control + melatonin SCIR	SCIR + melatonin (50 mg/kg, i.p.)	10 min before ischemia occlusion	ND	Melatonin decreases the activity of CASP3 and reduces the formation of MDA in SCI animals	Aydemir et al. (2016)
							Melatonin decelerates the loss of GSH	
38	New Zealand rabbit (M, 2000–2,500 g)	Infrarenal aorta temporarily occluded	Sham SCIR	SCIR + melatonin (10 mg/kg, i.v.)	10 min before ischemia occlusion	Modified Tarlov (6, 24, 48 hpi)	Melatonin treatment improves the neurologic function	Korkmaz et al. (2008)
							Melatonin reduces oxidative stress indicated by the decreased tissue and serum MDA levels and the increased tissue and serum GSH level compared to the SCI group	
							Melatonin attenuates ischemia-induced necrosis	
39	New Zealand rabbit (M, 2,800–3,500 g)	Infrarenal aorta temporarily occluded	SCIR	SCIR + melatonin (10 mg/kg, i.p.)	(a) 10 min before or (b) 10 min after ischemia occlusion	ND	Both melatonin preconditioning and post-ischemia treatment reduce ischemia-induced oxidative damage in the spinal cord, indicated by the decrease CAT and GSH-Px levels and increase SOD content	Erten et al. (2003)
40	Wistar rat (M, 180–220 g)	C1–T2 irradiation	Control control + melatonin SCI	SCI + melatonin (first dose 100 mg/kg, following dose 5 mg/kg, i.p.)	30 mpi (100 mg/kg) and qd (5 mg/kg) throughout the experiment	Frequency and onset of myelopathy Qd for 350 dpi	Melatonin treatment downregulates the VEGF expression at 20th and 22nd weeks after irradiation	Haddadi et al. (2013)
							Melatonin-treated irradiated rats show a longer latency period for radiation-induced hind limb paralysis and weakness compared to the irradiated rats	
41	Wistar rat (M, 180–220 g)	C1–T2 irradiation	Control control + melatonin SCI	SCI + melatonin (100 mg/kg, po. and then 5 mg/kg, p.o.)	30 mpi (100 mg/kg) and then qd (5 mg/kg)	ND	Orally melatonin administration inhibits the expression of TNF-α and decreases the MDA level	Haddadi and Fardid (2015)
42	SD rat (F, 250–300 g)	T8–9 photo thrombosis	Sham SCI + V	SCI + melatonin (50 mg/kg, i.p.)	1 hpi and bid for 7 dpi	BBB (3, 7,14, 21, and 28 dpi)	Melatonin improves functional outcome indicated by an increased BBB score	Piao et al. (2014)
							Melatonin reduces spinal cord edema by decreasing the expression of MMP2 and MMP9	

BMS, Basso Mouse Scale; BBB, Basso–Beattie–Bresnahan score; BSCB, blood–spinal cord barrier; bid, twice a day; DD, 24 h constant dark; F, female; i. p., intraperitoneal; i. v., intravenously; L/D, 12/12 h light/dark; LL, 24 h constant light; M, male; min, minutes; (m/h/d)pi, (minute/hour/day) post injury; MP, methylprednisolone; ND, not described; KO, knock-out; p. o., peros; qd, once a day; ROS, reactive oxygen species; SCI, spinal cord injury; SCIR, spinal cord ischemia–reperfusion injury; SD rats, Sprague Dawley rats; s. c., subcutaneously; V, vehicle; WT, wild type.

### 3.1 Effects of melatonin in spinal cord injury

#### 3.1.1 Melatonin maintains the integrity of the blood–spinal cord barrier and decreases edema

The blood–spinal cord barrier (BSCB) consists of vascular endothelial cells, astrocytes, pericytes, and basement membrane. It regulates molecular exchange between blood and the spinal cord and plays an important role in maintaining the fluid microenvironment of the spinal cord (Haddadi et al., 2013). The compromised integrity of the BSCB increases its permeability to plasma components, e.g., neutrophil infiltration happens at the site of injury following traumatic SCI, leading to vasogenic spinal cord edema (Wu et al., 2014). In traumatic SCI, the BSCB permeability is increased quickly within 48 h post SCI; the melatonin treatment maintains the integrity of the BSCB via regulating the tight junction proteins and protecting endothelial cells and pericytes (Wu et al., 2014). Jing et al. (2016) showed that melatonin attenuates edema by decreasing the expression of aquaporin 4 (AQP4) and intercellular cell adhesion molecule-1 (ICAM1), which restrains microvessel loss and sustains microcirculation (Wu et al., 2014). In the irradiated spinal cord tissue, the mRNA expression of the vascular endothelial growth factor (VEGF), which is critical in BSCB destruction (Haddadi et al., 2013), is increased at 20th and 22nd week after irradiation (Haddadi et al., 2013). Melatonin preconditioning decreases the expression of VEGF and significantly reduces vasodilation, congestion, and cavitation in the white matter, which promotes functional recovery in the irradiated animals (Haddadi et al., 2013).

#### 3.1.2 Melatonin prevents neural death and facilitates neuroplasticity

Neurons are the structural and functional units of the nervous system, while glial cells support neurons. Neurons communicate to one another based on a synaptic structure. Oligodendrocytes form the myelin sheath around axons, which promises an efficient long-distance impulse transmission from neuronal soma to the presynaptic membrane. In SCI, the deteriorated microenvironment after SCI jeopardizes neuronal vitality and causes neuronal death. Wang Q. et al. (2022) found that melatonin inhibits neuronal death at the acute phases of SCI and promotes rehabilitation afterward by attenuating oxidative stress and preventing mitochondrial dysfunction (Wang H. et al., 2022). Moreover, intrathecal melatonin administration significantly preserves the myelinated areas and increases the number of sensory neurons in the white matter after SCI (Fakhri et al., 2021). In a word, melatonin administration prevents axonal degeneration and demyelination (Majidpoor et al., 2020a; Majidpoor et al., 2020b). Parallelly, the endogenous melatonin content in the rostrocaudal region of the spinal cord is increased by a 12-h light/12-h dark cycle, promoting oligodendrogenesis and excitatory synaptic formation (Hong et al., 2019). Neuroplasticity drives functional restoration in SCI. Melatonin facilitates neuronal remodeling by increasing the expression of the brain-derived neurotrophic factor (BDNF) and growth-associated protein-43 (GAP-43) (Jing et al., 2017). Bi et al. (2021) found that melatonin administration after SCI significantly increases the expression of GAP-43, synaptic protein 1 (SYN1), neurofilament 200 (NF200), and postsynaptic density protein 95 (PSD-95) (Bi et al., 2021).

#### 3.1.3 Melatonin decreases scar formation and promotes axon regeneration

Reactive astrogliosis forms the core of fibrotic scar at the SCI lesion site. The glial scar, a dense limiting border outside the injury site, is the primary cause of the failure in axonal regeneration, and reducing glial scar formation after SCI contributes to the regrowth of transected axons (Krityakiarana et al., 2016). It has been reported that melatonin suppresses the proliferation and accumulation of astrocytes (Yang et al., 2020). Krityakiarana et al. (2016) reported that melatonin reduces inflammatory response and scar formation, which promotes axonal outgrowth (Krityakiarana et al., 2016). Moreover, Hong et al. (2019) reported that the melatonin precursor N-acetylserotonin (NAS) activates the tropomyosin receptor kinase B (TRKB)-induced protein kinase B (AKT)–extracellular-regulated kinase (ERK) signaling cascade, which facilitates axonal growth after SCI (Hong et al., 2019).

### 3.2 Mechanisms underlying melatonin facilitating functional recovery in spinal cord injury

#### 3.2.1 Melatonin attenuates oxidative stress

Melatonin is an effective free-radical scavenger, sustaining the balance between reduced glutathione (GSH) and oxidized glutathione disulfide (GSSG) and preventing excess lipid peroxidation in the aged mouse brain (Carretero et al., 2009). In traumatic and ischemia/reperfusion SCI, melatonin corrects the level of malondialdehyde (MDA), GSH, superoxide dismutase (SOD), nitrite oxide (NO), xanthine oxidase (XO), thiobarbituric acid reactive substance (TBARS), inducible nitric oxide synthase (iNOS), and myeloperoxidase (MPO), which benefits rehabilitation (Fujimoto et al., 2000; Kaptanoglu et al., 2000; Taskiran et al., 2000; Erten et al., 2003; Liu et al., 2004; Song et al., 2013). Moreover, Gül et al. (2005) revealed a dose-dependent effect of exogenous melatonin on protecting the axons and myelin in white matter at 24 h after SCI, but not on preventing early lipid peroxidation (Gül et al., 2005). Interestingly, Park et al. (2012) reported a loss of efficacy of melatonin treatment in decreasing the mRNA expression of iNOS and accelerating motor function recovery when exposing SCI rats to 24-h constant light, which suggests the significance of endogenous melatonin and melatonin rhythm in functional recovery in SCI (Park et al., 2012).

#### 3.2.2 Melatonin reduces inflammation

ROS functions as a critical second messenger that modulates inflammation through redox-sensitive mechanisms. Melatonin works as an antioxidant, attenuating neuroinflammation by altering the oxidative stress-induced gene expression profiles in SCI, such as peroxisome proliferator-activated receptors α (PPAR-α), toll-like receptor-4 (TLR4), nuclear factor kappa B (NF-κB), and NRF2. Specifically, melatonin exhibits anti-inflammatory effects against the secondary injury in wild-type mice by binding to PPAR-α, indicated by a loss of response to melatonin in the PPAR-α knockout (KO) mice (Paterniti et al., 2017). Moreover, melatonin administration during the acute phase after SCI suppresses inflammation through inhibiting the TLR4/NF-κB signaling pathway (Majidpoor et al., 2020a). Recently, Wang Q. et al. (2022) reported that melatonin reduces the generation of nucleotide-binding domain-like receptor protein 3 (NLRP3) inflammasomes via activating NRF2 and downstream antioxidant responsive element (ARE) signaling transduction, which reduces mitochondrial dysfunction and accelerates neurological functional recovery (Wang H. et al., 2022).

M1 microglia (pro-inflammatory) is a main source of tumor necrosis factor alpha (TNF-α), interferon-γ (INF-γ), and interleukin-1β (IL-1β) in CNS. Haddadi and Fardid (2015) found that the TNF-α expression increases within hours in the radiation-injured spinal cord tissue, while oral melatonin administration during the acute phase inhibits TNF-α and aids in SCI (Haddadi and Fardid, 2015). The effectiveness of melatonin in diminishing pro-inflammatory cytokines has been evidenced, such as INF-γ and IL-1β (Xu et al., 2018; Jing et al., 2019; Yang et al., 2020). Moreover, it has been reported that melatonin promotes microglia M2 polarization (anti-inflammatory type) rather than M1 (Zhang et al., 2019).

### 3.3 Melatonin inhibits cellular apoptosis

Oxidative stress is a potent apoptotic inducer, which disrupts Ca^2+^ homeostasis, causes mitochondrial dysfunction, e.g., mtDNA damage and cytochrome c release, and induces typical apoptotic proteins, e.g., the increased expression of cleaved caspase-3 (CASP3) and B-cell lymphoma 2 (BCL2) and the decrease in the bcl-2-like protein 4 (BAX) content (Aydemir et al., 2016). Aydemir et al. (2016) found that melatonin deficiency impedes sensory and motor function recovery in the ischemic spinal cord injured rats, while pretreatment with melatonin alleviates neuronal apoptosis and accelerates functional recovery by activating the phosphoinositide 3-kinase (PI_3_K)–akt serine/threonine kinase 1 (AKT1) signaling pathway (Aydemir et al., 2016). Parallelly, the administration of melatonin during the first 3 days after traumatic SCI ameliorates neuronal apoptosis via provoking the wingless–int (Wnt)/β-catenin signaling transduction (Shen et al., 2017).

Oxidative stress damages cellular constituents, e.g., proteins, DNA, and lipids, which turns on the autophagic process (Li et al., 2019). The mechanistic target of rapamycin (mTOR) is a pharmacologic target for autophagy regulation. It has been found that melatonin enhances autophagy and inhibits neuronal apoptosis after SCI through activating the PI_3_K/AKT/mTOR signaling pathway (Li et al., 2019). In addition, mTOR, 5′AMP-activated protein kinase (AMPK), and NAD-dependent deacetylase sirtuin-1 (SIRT1) interplay in regulating metabolic stress. Gao T. et al. (2020) indicated that melatonin exerts neuroprotection via activating autophagy through the SIRT1/AMPK signaling pathway (Gao K. et al., 2020).

## 4 Melatonin maintains the function of peripheral organs and aids in spinal cord injury

SCI causes functional disorders in the peripheral organs, including the gastrointestinal system (Zhang C. et al., 2018), kidney (Akakin et al., 2013), bladder (Erşahin et al., 2012), testes (Yuan et al., 2016), and corpus cavernosum (Tavukçu et al., 2014), which is attributed to the damaged autonomic innervation by the sympathetic and parasympathetic fibers derived from the spinal cord (Table 3). For example, the damaged autonomic nervous system loses gastrointestinal sympathetic control, leading to insufficient intestinal motility and deficient mucosal secretions (Zhang C. et al., 2018). The gut microbiota works as a virtual endocrine organ, regulating mood and behaviors via the microbiota–gut–brain axis and immunity via the intestinal neuro-immune axis (Jacobson et al., 2021; Huang et al., 2022). Hence, SCI affects the behavior of the gastrointestinal system, alters the constitution of gut microbial community, and causes systemic disorders. The alteration in gut microbiota is associated with the metabolite profiles in SCI (Kang et al., 2023). Gut dysbiosis impairs rehabilitation in SCI (Agahi et al., 2018). The gut microbiota–brain axis mediates the psychological stress after SCI (Musleh-Vega et al., 2022). Fecal microbiota transplantation exerts neuroprotective effects on SCI mice via modulating the microenvironment at the lesion site (Jing et al., 2022). Furthermore, melatonin mitigates the microbiota dysbiosis in the segment of the jejunum in the short-term sleep-deprived mice (Gao T. et al., 2020). Jing et al. (2019) showed that melatonin treatment reduces the relative abundance of *Clostridium perfringens* and increases that of *Lactobacillus*, which attenuates SCI-induced dysbiosis (Jing et al., 2019). Moreover, melatonin treatment improves the function of bladder (Erşahin et al., 2012), decreases the SCI-increased permeability of capillaries, and increases the SCI-reduced testicular blood flow (Yuan et al., 2016) by coping with oxidative stress.

**TABLE 3:** Non-comprehensive review of melatonin protecting peripheral organs in experimental spinal cord injury.

No.	Animal (gender/weight)	Model	Control	Treatment	Melatonin (dosage/duration)	Assessment	Major findings	Ref.
1	Wistar rat (M, 250–300 g)	T7–10 weight-drop	Sham + V SCI + V	SCI + melatonin (10 mg/kg, i.p.)	0 hpi and qd for 7 dpi	Motor function score (7 dpi)	Melatonin restrains the loss of motor functions compared to the SCI group	Akakin et al. (2013)
							Melatonin prevents oxidative damage to the kidneys, indicated by the decreased MDA level and MPO activity and the increase in GSH concentration	
							Melatonin prevents the degeneration of renal tissue, indicated by the decrease in the percentage of degenerated glomeruli in semi-quantitative histopathological scoring	
2	Wistar rat (ND, 250–300 g	T7–10 weight-drop	Sham SCI + V	SCI + melatonin (10 mg/kg, i.p.)	15 mpi	Motor function score (7 dpi)	Melatonin improves the motor function	Erşahin et al. (2012)
							Melatonin decreases oxidative stress in spinal cord and urinary bladder indicated by the decreased MDA concentration and the increased GSH level	
							Melatonin decreases the activity of CASP3 and increases the expression of NGF in the spinal cord and bladder compared to the SCI rats	
3	C57BL/6 mouse (M, ND)	T10 weight-drop	Sham SCI + V	SCI + melatonin (10 mg/kg, i.p.)	30 mpi	ND	Melatonin treatment protects the testes, attenuating the SCI-induced increased capillary permeability	Yuan et al. (2016)
							Melatonin reduces neutrophil infiltration, decreases MDA and MPO levels, and increases the GSH/GSSG ratio	
4	Wistar rat (ND, 250–300 g)	T7–10 weight-drop	Sham SCI tadalafil	SCI + melatonin (10 mg/kg, i.p.) SCI + melatonin (10 mg/kg, i.p.) +tadalafil	0 hpi and qd for 7 dpi	Ratio of intracavernosal pressure to mean arterial pressure (7 dpi)	Melatonin protects cavernosum tissues and restores erectile function	Tavukçu et al. (2014)
							Melatonin reverses the oxidative damage indicated by the decreased MPO and CASP3 levels, increased GSH and NGF contents, and increased SOD activity in cavernosum tissues compared to the SCI group	
5	C57BL/6 mouse (F, 18–22 g)	T10 weight-drop	Sham sham + mealtonin SCI + V	SCI + melatonin (10 mg/kg, i.p.)	Bid (07:00, 19:00) for 42 dpi	BMS and subscore (3, 7, 14, 21, and 28 dpi)	Melatonin improves locomotor recovery in SCI mouse	Jing et al. (2019)
							Melatonin maintains intestinal barrier integrity, accelerates gastrointestinal transit, and reshapes gut bacterial composition	
							Melatonin alters metabolic profiling and downregulates expression of pro-inflammatory cytokines in SCI mouse	

Bid, twice a day; F, female; i. p., intraperitoneal; M, male, (m/h/d)pi, (minute/hour/day) post injury; MP, methylprednisolone; ND, not described; qd, once a day; ROS, reactive oxygen species; SCI, spinal cord injury; V, vehicle.

## 5 Synergistic effects of melatonin

Based on the bulk reports regarding melatonin’s protective roles in SCI, the synergistic effects of melatonin are explored (Table 4), including interacting other medications, functioning as a supplement in rehabilitation training, and working as a preconditioning stimulus in stem cell therapy. Moreover, melatonin has been employed by biomedical material engineering as an antioxidant agent in treating SCI and a differentiation signal to activate the endogenous neural cells.

**TABLE 4:** Non-comprehensive review of the synergistic effects of melatonin in spinal cord injury.

No.	Animal (gender/weight)	Model	Control	Treatment	Melatonin (dosage/duration)	Assessment	Major findings	Ref.
1	Wistar rat (F, 200–250 g)	T7–10 weight-drop	Sham SCI + MP SCI + V	SCI + melatonin (10 mg/kg, i.p.) SCI + MP + melatonin (10 mg/kg, i.p.)	4 and 24 hpi and 10 dpi	Motor function scale, inclined plane test (1, 3, 5, 7, and 10 dpi)	Melatonin improves locomotor recovery	Cayli et al. (2004)
							Melatonin decreases the MDA activity at 4 and 24 hpi and 10 dpi	
							Melatonin combines with the MP results in a cumulative effect on decreasing lipid peroxidation during the subacute phase but not enhances neurological recovery	
2	SD rat (ND, ∼250 g)	T9 weight-drop	Control SCI + MP SCI + HMP	SCI + melatonin (15 mg/kg, i.p) SCI + melatonin (15 mg/kg, i.p)+HMP	ND	ND	Melatonin decreases apoptotic nerve cells and Nissl bodies loss in SCI	Bi et al. (2022)
							Melatonin reduces the standard dose of MP applied in SCI and limits the adverse effects of MP	
							Melatonin synergizes with HMP, decreases the number of apoptotic nerve cells via activating the PI_3_K/AKT1 pathway, and promotes axon outgrowth	
3	Mice (ND)	T6-7 clamp compression	Sham + V sham + melatonin + DEX SCI + V SCI + DEX	SCI + melatonin (10 mg/kg, i.p.) SCI + melatonin (10 mg/kg, i.p.) +DEX	1 and 4 hpi	BBB (Qd for 10 days)	Melatonin combined with DEX improves motor recovery	Genovese et al. (2007)
							Melatonin combined with DEX decreases edema, neutrophil infiltration, and apoptosis in the spinal cord	
							Melatonin combined with DEX reduces the effective concentration of DEX and restrains its side effects	
							No anti-inflammatory effect is observed when either melatonin or DEX is administrated alone	
4	New Zealand rabbit (M, 2,150–2,800 g)	Infrarenal aorta temporarily occluded	Sham SCIR SCIR + zinc	SCIR + melatonin (10 mg/kg, i.p.) SCIR + zinc + melatonin (10 mg/kg, i.p.)	0 hpi	Tarlov (1, 8, and 24 hpi)	Melatonin and zinc increases the neurological score of SCIR rats and improves their histopathological situation	Kalkan et al. (2007)
							Melatonin and zinc decrease the number of apoptotic cells and increase that of the intact ganglion cells	
							Melatonin and zinc decrease the formation of MDA and increase the activity of GPx	
5	SD rat (M, 250–270 g)	T9–10 weight-drop	SCI	SCI + melatonin (10 mg/kg, s.c.) SCI + melatonin (10 mg/kg, s.c.)+exercise	Bid for 21 dpi	BBB (1, 3, 7, 14, and 21 dpi)	Melatonin supplied with exercise improves the hind limb function	Lee et al. (2014)
							Melatonin synergizes with physical exercise, stimulates endogenous neural stem/progenitor cells proliferation, and facilitates neural differentiation into neurons and glial cells	
6	SD rat (M, 230–270 g)	T9–10 weight-drop	Control SCI + exercise	SCI + melatonin (10 mg/kg, s.c.) + exercise	Bid for 42 dpi (07:00, 19:00)	BBB (1, 3, 7, 14, 21, and 28 dpi)	Melatonin combined with exercise significantly increases locomotor fucntion, reduces iNOS mRNA expression, and increases motor neuron number in the ventral horn compared to exercise alone	Park et al. (2010)
							Protein expressions of BECN1, LC3, p53, and IKKa are similar among the melatonin combined exercise SCI group, exercise SCI group, and SCI group	
7	SD rat (M, 250–300 g)	T9–10 clamp compression	Control sham SCI + V SCI + ADSCs	SCI + melatonin-ADSCs (5 μM melatonin pretreated ADSCs, i.v.)	Qd for 7 dpi	BBB (7, 14, 21, 28, 35, 42, 49, and 56 dpi)	Melatonin pretreatment enhances the number of engrafted cells and neuronal differentiation (neurons, astrocytes, and oligodendrocyte lineage cells)	Naeimi et al. (2022)
							Functional improvement is not detected in the melatonin-pretreated ADSCs group compared to ADSCs	
8	WT C57BL/6 mouse (ND)	T8 weight-drop	SCI + PBS SCI + EVs SCI + shNC-MEVs	SCI + MEVs SCI + shUSP29-MEVs (200 μg of total extracellular vesicles protein, iv)	0 hpi	BMS (1, 3, 7, 14, 21, and 28 dpi)	MEV administration accelerates motor behavioral recovery compared to EVs. MEVs facilitate microglia/macrophages polarization to the M2 subtype in WT mouse in SCI via stabilizing NRF2, indicated by a loss of response to MEVs in NRF2 KO mouse. MEVs deliver USP29 to microglia/macrophage and promote M2 polarization, while the MEVs obtained from the shUSP29-treated MSCs do not affect microglia/macrophage polarization. Melatonin reduces global N6-methyladenosine (m^6^A) modification and expression of the m^6^A “writer” METTL3 in MSCs, which stabilizes the USP29 mRNA	Liu et al. (2021)
	Nrf2 KO C57BL/6 mouse (ND)		SCI + EVs	SCI + MEVs				
9	Kunming mouse (ND, 40–45 g)	T6–10 weight-drop	SCI + PBS SCI + AECs	SCI + neural cells 1 μM melatonin to induce AEC formation of neural cells	AEC neural differentiation	BBB (3 dpi and once 3 days for 6 weeks)	Transplantation of AECs prtreated with melatonin and WNT4 leads to a significant increase in locomotor recovery	Gao et al. (2016)
							Melatonin cooperated with WNT4 increases the vitality of the bovine AECs, which facilitate cell survival in stem cell transplantation in SCI	
							Melatonin promotes the differentiation of bovine AECs into neural cells by stimulating MT_1_	
10	SD rat (ND)	T9 clamp compression	SCI + Lap/MS	SCI + melatonin (5 mg/kg, i.v.) SCI + MS@Melatonin (5 mg/kg, i.v.) SCI + Lap/MS@Melatonin (5 mg/kg, i.v.)	0 hpi and qd for 7 dpi	BBB (1, 3, 7, 14, and 28 dpi)	Melatonin administration reduces neuronal apoptosis indicated by the decreased CASP3 expression	Zhang et al. (2021)
			SCI + PM/MS	SCI + melatonin (5 mg/kg, i.v.) SCI + MS@Melatonin (5 mg/kg, i.v.) SCI + PM/MS@Melatonin (5 mg/kg, i.v.)		BBB (1, 3, 7, and 14 dpi)	Lap/MS@Melatonin promises a stable and prolonged melatonin release	
							PM/MS@Melatonin realizes precision-targeted delivery of melatonin intravenously in SCI	
							Drug delivery system improves the motor function recovery post SCI via regulating the macrophage/microglia polarization	
							Drug delivery system restrains the oxidative stress, inflammatory response, and cellular apoptosis after SCI	

ADSCs, adipose-derived stem cells; AECs, amniotic epithelial cells; BBB, Basso–Beattie–Bresnahan score; bid, twice a day; DEX, dexamethasone; EVs, extracellular vesicles derived from mesenchymal stem cells without pretreatment with melatonin; F, female; (H)MP, (half dose of) methylprednisolone; i. p., intraperitoneal; i. v., intravenously; MEVs, extracellular vesicles derived from mesenchymal stem cells pretreated with melatonin; Lap/MS@Melatonin, poly (lactic-co-glycolic acid) (PLGA) MS loaded with melatonin and mixed further with Laponite hydrogels; M, male, MS, sustained-release microspheres; (h/d) pi, (hour/day) post injury; MPSS, methylprednisolone sodium succinate; ND, not described; KO, knock-out; qd, once a day; ROS, reactive oxygen species; SCI, spinal cord injury; SCIR, spinal cord ischemia–reperfusion injury; SD, rats, Sprague Dawley rats; s. c., subcutaneously; (sh)USP29, (knockdown) ubiquitin-specific protease 29; V, vehicle; WT, wild type.

### 5.1 Melatonin administrated with other drugs

Methylprednisolone (MP) is a corticosteroid medication applied during the acute phase in SCI (Cayli et al., 2004). Cayli et al. (2004) reported that combining melatonin with MP results in a better neurological recovery during the subacute phase after SCI by inhibiting the accumulation of lipid peroxidation (Cayli et al., 2004). Moreover, melatonin administrated with the corticosteroid dexamethasone (DEX) significantly reduces tissue damage and promotes motor function recovery via reducing apoptosis and polymorphonuclear leukocyte infiltration indicated by the downregulated protein expressions of TNF-α and iNOS in the spinal cord (Genovese et al., 2007). The combined administration elevates the curative effect of DEX, i.e., reducing to a 10th of the original effective concentration, and avoids its side effects, such as infection, osteonecrosis/osteoporosis, and depression (Genovese et al., 2007). Moreover, the combination therapy of melatonin and tadalafil (a medicine for erection problems) is more effective in restoring erectile function via attenuating the oxidative damage to cavernosum in SCI compared to the individual drugs (Tavukçu et al., 2014).

### 5.2 Melatonin supplements rehabilitation training

SCI disrupts the signaling transmission between the brain and body, and SCI rehabilitation exercise helps restore brain–body communication via facilitating neuronal regeneration in the injured spinal cord. Melatonin administration combined with motor-driven treadmill exercise suppresses neuroinflammatory response, reduces neuronal deformation, increases endogenous neural stem/progenitor cells, enhances neuronal differentiation, and improves the recovery of motor function after SCI (Park et al., 2010). In line with the previous report, Lee et al. (2014) found that the synergistic effect of melatonin combined with treadmill exercise. It creates a healing niche which facilitates proliferation and differentiation of endogenous neural stem/progenitor cells, promotes neuronal regeneration, and improves neurological function in SCI rats (Lee et al., 2014).

### 5.3 Melatonin serves in stem cell therapy

The melatonin precondition provides a promising extracellular vesicle-based or stem cell-based approach for treating SCI. It has been reported in mesenchymal stem cells (MSCs) that melatonin reduces methyltransferase-like 3 (METTL3) expression and global N6-methyladenosine (m^6^A) modification, which stabilizes the mRNA of ubiquitin specific peptidase 29 (USP29) (Liu et al., 2021). Liu et al. (2021) harvested USP29-contained extracellular vesicles from the melatonin-pretreated MSCs (MEVs) (Liu et al., 2021). The USP29-contained MEVs transfer USP29 to microglia and macrophages in the injured site, which stabilizes NRF2, facilitates M2-like polarization, and consequently, promotes functional behavioral recovery in the SCI mice (Liu et al., 2021). Moreover, melatonin, working as an antioxidant, facilitates the vitality of the adipose-derived stem cells (ADSCs/adMSCs) *in vitro* before stem cell transplantation (Naeimi et al., 2022). The melatonin-pretreated ADSC/adMSC positively affects the engraftment and neuronal differentiation in SCI (Naeimi et al., 2022). Similarly, melatonin administrated with wnt family member 4 (WNT4) promotes neural cell differentiation of the bovine amniotic epithelial cells, and the neural cell transplantation has been proven to accelerate the motor function recovery in SCI (Gao et al., 2016).

### 5.4 Melatonin is employed by biomedical material engineering

Melatonin is employed in biomedical material engineering to act directly on the endogenous stem cells. Two new sustained-release melatonin delivery systems to prolong melatonin release and enhance melatonin’s role in facilitating M2 polarization are developed in consideration of the complicated conditions and significant inter-individual differences in SCI patients (Zhang et al., 2021). For *in situ* injection, an injectable Lap/MS@Melatonin micro-gel compound, i.e., poly (lactic-co-glycolic acid) (PLGA) sustained-release microspheres (MSs) loaded with melatonin mixed with Laponite hydrogels, has been designed (Zhang et al., 2021). To sustain and for precision-targeted delivery of melatonin intravenously, a nano-PM compound, namely, PM/MS@Melatonin, i.e., nanospheres coated with a platelet membrane (PM) loaded with melatonin, is designed (Zhang et al., 2021). Moreover, a 3D-printed scaffold for a controlled release of melatonin has been developed for curing long-range nerve defects (Qian et al., 2018). It has been found that this melatonin/polycaprolactone nerve guide conduit reduces oxidative stress, neuroinflammation, and nerve cell apoptosis; provides energy for nerves; facilitates nerve debris clearance; and stimulates neural proliferation (Qian et al., 2018).

## 6 Side effects of melatonin

Adverse events have been reported in melatonin treating sleep disorders, including excessive sedation (Panda et al., 2022), cognitive disorders (Besag et al., 2019), nocturnal hypotension (Grossman et al., 2011), and impaired glucose tolerance (Rubio-Sastre et al., 2014). The suggested dose of melatonin in clinic is 0.3–1.0 mg/day internationally. However, a clinical trial indicated no significant difference between melatonin and placebo arms in the rate of adverse events, even at a dose as high as 1,600 mg/day (Foley and Steel, 2019). Moreover, systemic reviews indicate no adverse events of life-threatening or of major clinical significance (Grossman et al., 2011; Besag et al., 2019; Panda et al., 2022). The bioavailability of exogenous melatonin is highly variable due to the first-pass metabolism and poor absorption, ranging from 1% to 37% (Andersen et al., 2016). Especially, in cirrhotic patients, the clearance of melatonin is slowed down (Chojnacki et al., 2013). Hence, caution should be exercised when administering melatonin with other drugs (Foster et al., 2015). For example, taking melatonin with sedatives might cause sleepiness and slow breathing (Panda et al., 2022). Furthermore, a case report indicated that a good initial response to melatonin was found at the beginning in treating intellectual disability and sleep disorders; the patients lost response to melatonin afterward potentially due to the delayed melatonin clearance (Braam et al., 2010).

To avoid the adverse effects, lowering effective concentration, increasing the effectiveness of melatonin treatment, and seeking melatonin analogs are considered. For example, the effect of β-methyl-6-chloromelatonin, which exerts a direct soporific effect in treating insomnia without changing in body temperature, heart rate, or blood pressure compared to melatonin (Zemlan et al., 2005), has been studied in SCI though no statistical significant outcome was found (Fee et al., 2010). Other novel synthetic analogs, mainly the melatonin derivatives, are under study, such as the 2-phenylindole derivatives (Suzen et al., 2006), 5-bromoindole derivatives (Gürkök et al., 2009), 2-indole aldehyde derivatives (Suzen et al., 2013), and 5-chloroindole derivatives (Yılmaz et al., 2012).

## 7 Conclusion and perspectives

**FIGURE 1:**
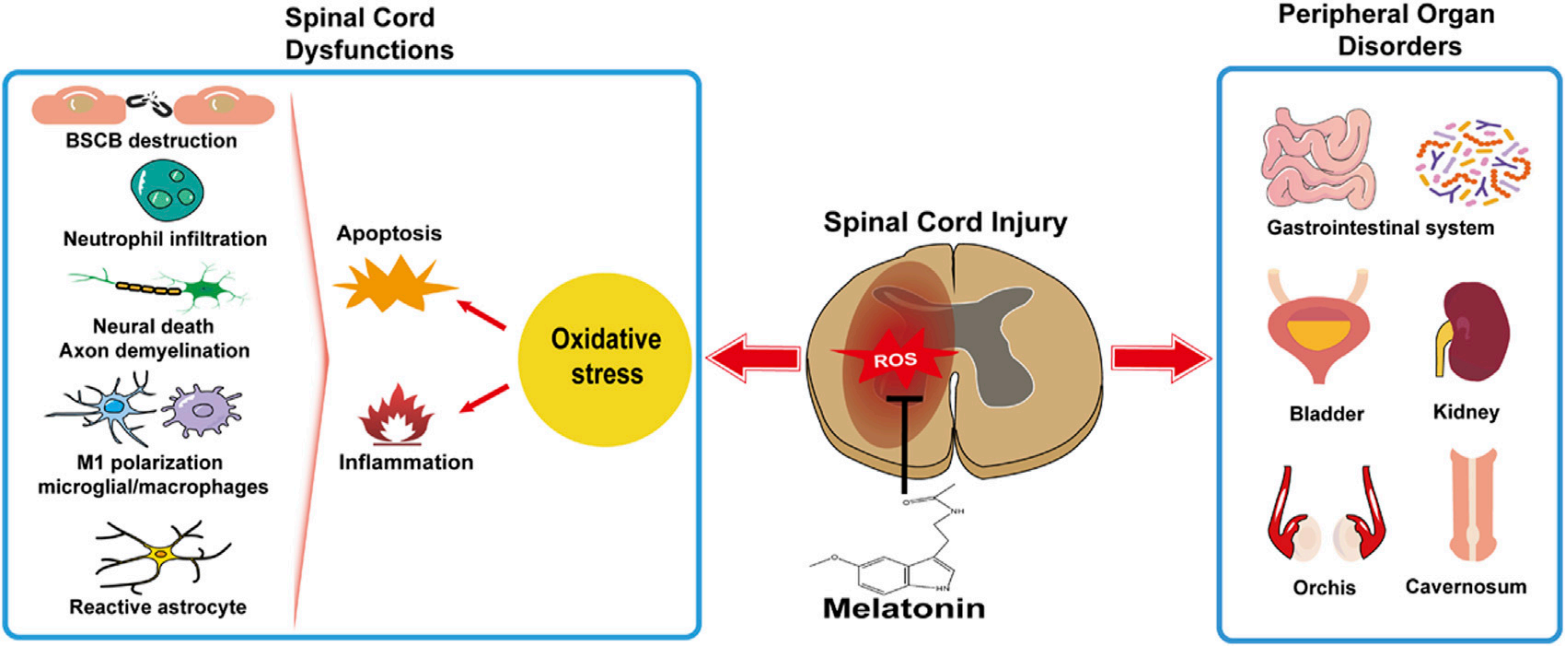
Melatonin facilitates systemic recovery by preventing spinal cord dysfunctions and peripheral organ disorders.

In this review, the relationship between the melatonin rhythm and SCI has been unveiled in clinic, and the feasibility of melatonin treatment in facilitating rehabilitation under the circumstances of various SCI degrees and segments has been verified in experimental SCI models. Oxidative stress is a primary cause in cytotoxicity and disease. Melatonin administrated independently or synergized with other medications, rehabilitation training, stem cell transplantation, and biomedical material engineering, as a natural antioxidant therapy, aids in SCI. Generally, melatonin copes with oxidative stress and mitigates subsequent neuroinflammation and cellular apoptosis in SCI, resulting in reduced neural death and debris, restored BSCB, attenuated edema, decreased scar formation, a better neuronal regeneration, and neuroplasticity and microcirculation in the injured spinal cord (Figure 1). Moreover, melatonin attenuates oxidative damage, prevents functional disorders in the peripheral organs, and has a beneficial influence on systemic recovery in SCI (Figure 1). Though the melatonin’s protective roles in preventing secondary injuries in SCI have been studied for decades and many signaling pathways are reported involved in melatonin facilitating functional recovery and attenuating tissue damage, the receptor-dependent mechanism is rarely mentioned, e.g., MT_1_ or MT_2_. Due to the potential rise of long-term melatonin exposure and the high dose of melatonin used in SCI [orally: 5 mg/kg, intraperitoneal injection: 5–100 mg/kg (reported in experimental SCI research, Table 2); compared to clinical dosage: physiological dose (0.3–1.0 mg/qd) and supraphysiological dose for occasional use (≥3 mg/qd), seeking melatonin analogs to prevent drug resistance and designing selective compound for an effective melatonergic therapy in SCI are advocated.
